# Tanshinone Ameliorates Glucocorticoid-Induced Bone Loss *via* Activation of AKT1 Signaling Pathway

**DOI:** 10.3389/fcell.2022.878433

**Published:** 2022-03-28

**Authors:** Yanjun Wang, Lin Liu, Zechao Qu, Dong Wang, Wangli Huang, Lingbo Kong, Liang Yan

**Affiliations:** Department of Spine Surgery, Honghui Hospital, Xi’an Jiao Tong University, Xi’an, China

**Keywords:** tanshinone, osteoporosis, AKT1, signaling, bone

## Abstract

**Purpose:** Osteoporosis, a common disorder especially prevalent in the postmenopausal women and the elderly, is becoming a worldwide public health problem. Osteoporosis can cause severe joint pain, fragility fractures, and other symptoms, which can seriously impair the daily lives of affected patients. Currently, no gold-standard drug is available that can completely cure osteoporosis. Tanshinone is a traditional Chinese medicine, which can exhibit multiple biological activities. It might also display a protective effect on osteoporosis. However, the molecular mechanism through which tanshinone can improve osteoporosis remain unclear. The objective of our study is to explore the underlying mechanism behind the protective actions of tanshinone.

**Methods:** The common KEGG pathways of tanshinone-targeted genes and osteoporosis were analyzed by using bioinformatics analysis. The bioinformatics analysis results were further validated both by *in vitro* and *in vivo* experiments.

**Results:** 21 common KEGG pathways were identified between osteoporosis and tanshinone-targeted genes. It was further found that tanshinone could induce expression of AKT1, promote the proliferation of MSCs, and ultimately suppress their apoptosis.

**Conclusion:** Taken together, our findings indicate that tanshinone can alleviate osteoporosis, its effect was potentially mediated through modulating AKT1 expression. Thus, tanshinone could serve as a promising treatment option for osteoporosis.

## Introduction

Osteoporosis is a major bone-turnover disorder which can commonly affect the elderly and postmenopausal women (Chang et al., 2016). Although the “bone density” which reflects bone mass is an important quantitative indicator when diagnosing osteoporosis, the critical significance of osteoporosis in the clinical practice is that, this population is prone to fractures, leading to both increased disability and death. In addition to bone mass, bone quality and other non-skeletal factors can also be considered as potential risk factors for fractures (Martin and Correa, 2010).

Osteoporosis is primarily an age-related bone disease, which has emerged as a worldwide public health problem (Rajasree Vijayakumar, 2016). It has been reported that more than 9.9 million Americans suffer from osteoporosis (Wright et al., 2014). The incidence of fragility fractures is 50% in women and 20% in men (Cummings and Melton, 2002). The recurrence incidence was reported to be 2.12% in the first year, and 4.66% in the first 2 years (Ruan et al., 2011). Treatment and care after the fractures can cause heavy economic and spiritual burdens on the family. Statistical data has revealed that within 1 year after the hip fractures, approximately 20% of patients died with various complications, about 50% were disabled, and the quality of life was significantly reduced (Lin et al., 2014). Osteoporosis often can exhibit the symptoms of joint pain. At the same time, the patients with degenerative joint disease are often affected with osteoporosis. Osteoporosis and osteoarthropathy can arise as a result of certain potential shared mechanisms (Hart et al., 1994; Shen et al., 2013; Im and Kim, 2014; Geusens and van den Bergh, 2016).

There are currently no treatment modalities that can be used for the complete cure of osteoporosis. There are mainly two types of drugs used for the management of osteoporosis: bone formation promotors and resorption inhibitors. Most of the existing clinical drugs are bone resorption inhibitors. Hence, we need to identify and develop novel drugs, which can promote bone formation. Tanshinone, a traditional Chinese medicine, has been used clinically as a drug for the treatment of various chronic diseases (Kim et al., 2016; Li et al., 2016). Moreover, previous studies have shown that tanshinone can also exhibit the potential of both decreasing apoptosis of osteoblasts and promoting osteogenesis (Kim et al., 2004; Baker et al., 2015; Cheng et al., 2018; Zhu et al., 2018; Zhang et al., 2020). Bioinformatics analysis has been used in some previous studies to explore the mechanism of dugs or formulations on the various diseases (Mi et al., 2020a; Xiong et al., 2020a). Therefore, this research aimed to investigate the underlying mechanism of tanshinone in the treatment of osteoporosis through using bioinformatics analysis, followed by verification of the findings through some laboratory experiments.

## Materials and Methods

### Tanshinone-Targeted Genes Interaction Network Construction

Tanshinone-targeted genes were obtained from Search Tool for Interacting Chemicals (STITCH). The interaction network of tanshinone and its targeted genes were constructed using STITCH online tool. The closeness, betweenness, and the degree of the network genes were analyzed by Cytoscape 3.7.2. (Szklarczyk et al., 2016).

### Common KEGG Pathways of Osteoporosis and Tanshinone-Targeted Genes

Human osteoporosis KEGG pathways were retrieved from miRWalk2.0 (Dweep and Gretz, 2015). Database for Annotation, Visualization, and Integrated Discovery (DAVID) was used to generate the tanshinone-targeted genes enriched KEGG pathways with *p* < 0.05 (Huang da et al., 2009a; Huang da et al., 2009b). Thereafter, the common KEGG pathways of tanshinone-targeted genes and osteoporosis were identified by Venn Diagram (http://bioinformatics.psb.ugent.be/webtools/Venn/).

### Identification of the Hub Genes

The enrichment result of the top five KEGG pathways were generated and visualized in GO plot (Walter et al., 2015). The common genes of all the five KEGG pathways were selected as hub genes. Thereafter, we employed circlize R package, positions on the chromosome and degree centrality information of all the tanshinone-targeted genes were visualized (Gu et al., 2014).

### Identification of the KEGG Pathways Related to Tanshinone-Targeted Genes

The top five common KEGG pathways with the smallest *p* values were analyzed. We used Pathway Builder Tool 20 (www.proteinlounge.com) to retrieve the pathway part related to the tanshinone-targeted genes.

### Animals

The *in vivo* study was reviewed and approved by The Honghui Hospital, Xi’an Jiao Tong University Standing Committee on Animals. For our study, 30 female C57BL/6 mice (6-week-old) were obtained from the Department of Research of Honghui Hospital, Xi’an Jiao Tong University. To generate the murine osteoporosis model, eight-week-old male C57BL/6 mice were treated with a daily intragastric administration of prednisone acetate (2.1 mg/kg/d), and then, the 30 osteoporosis mice were distributed equally into the PBS treated group (*n* = 10), 10 mg/kg Tanshinone treated group (*n* = 10), and 30 mg/kg Tanshinone treated group (*n* = 10). We administered the treatment for 8 weeks.

### Cell Culture

MSCs were kindly donated by the Xi’an Jiao Tong University, Xi’an, China. Cells were cultured in a specific osteogenic differentiation medium at 37°C in a 5% CO_2_ incubator. Then, 1 μM dexamethasone was added to produce the *in vitro* osteoporosis model. After which they were designated as osteoporosis group, 0.2 nM Tanshinone group, and 0.6 nM Tanshinone group for 7 days.

### Micro-CT Scanning and Analysis

The micro-CT device used for analysis was BRUKER SkyScan 1276 scanner. The trabecular bone from 0.2 to 0.5 mm below the proximal tibia growth plate was measured. Finally, the total volume (TV), bone volume (BV), BV/TV, and bone mineral density (BMD) were calculated and analyzed.

### qRT-PCR Analysis

Thermal Cycler C-1000 Touch system (#10021377, Bio-Rad CFX Manager, United States) was used to perform qRT-PCR reactions after the total RNA extraction. The primers used in this study have been listed in Table 1.

**TABLE 1:** The primers used in the experiments.

Gene name	Primer sequence
hsa—Bcl-2—Forward	GCT​ACC​GTC​GTG​ACT​TCG​C
hsa—Bcl-2—Reverse	CCC​CAC​CGA​ACT​CAA​AGA​AGG
hsa—Bax—Forward	AGA​CAG​GGG​CCT​TTT​TGC​TAC
hsa—Bax—Reverse	AAT​TCG​CCG​GAG​ACA​CTC​G
hsa—Cyclin D1—Forward	GCG​TAC​CCT​GAC​ACC​AAT​CTC
hsa—Cyclin D1—Reverse	ACT​TGA​AGT​AAG​ATA​CGG​AGG​GC
hsa—Cyclin D3—Forward	TGC​GTG​CAA​AAG​GAG​ATC​AAG
hsa—Cyclin D3—Reverse	GGA​CAG​GTA​GCG​ATC​CAG​GT
hsa—GAPDH—Forward	AGG​TCG​GTG​TGA​ACG​GAT​TTG
hsa—GAPDH—Reverse	GGG​GTC​GTT​GAT​GGC​AAC​A

### Statistical Analysis

GraphPad Prism 8.0 was used for the statistical analysis. Statistical analysis was performed using Student’s t test (two groups) or ANOVA with Tukey’s post hoc test (over two groups).

## Results

### Tanshinone-Targeted Genes and the Interaction Network

**FIGURE 1:**
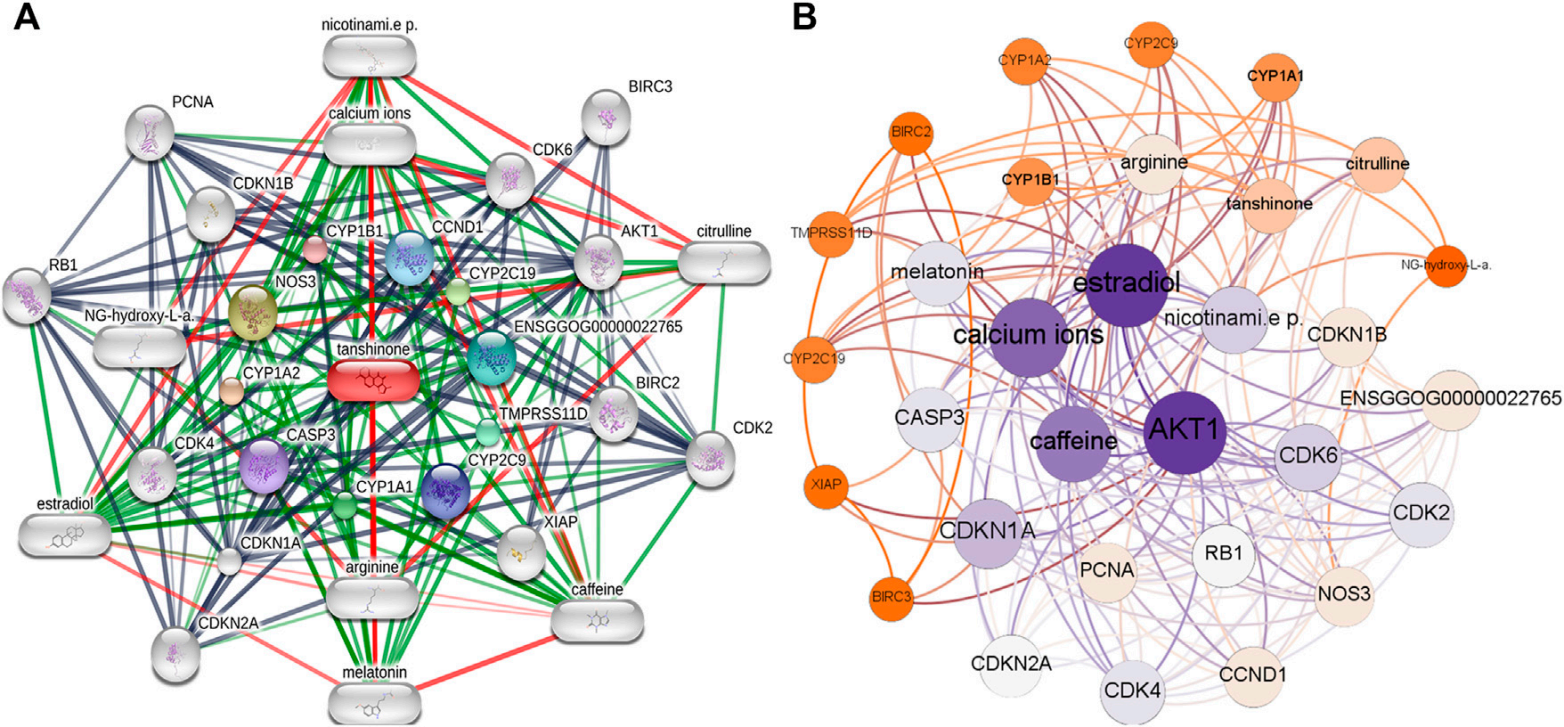
Interaction network of the Tanshinone-targeted genes. **(A)** Interaction network established by STITCH. **(B)** Weighted interaction network demonstrated by Gephi.

We retrieved 21 tanshinone-targeted genes using STITCH with the limitation of three shells. Thereafter, the interaction of tanshinone-targeted genes was established in STITCH (Figure 1A). *CCND1*, *CYP2C19*, *ENSGGOG00000022765*, *TMPRSS11D*, *CYP2C9*, *CYP1A1*, *CASP3*, *CYP1A2*, *NOS3*, *CYP1B1* were shown in the first shell which indicated that tanshinone potentially has a direct effect on these diverse genes. *CDK6*, *AKT1*, *BIRC2*, *XIAP*, arginine, *CDKN1A*, *CDK4*, *N*G-hydroxy-L-a, *CDKN1B*, calcium ions were shown in the second shell and *BIRC3*, citruline, *CDK2*, caffeine, melatonin, *CDKN2A*, estradiol, *RB1*, *PCNA*, nicotinami.e p. were shown in the third shell, which indicated that tanshinone might have a secondary effect on these genes. According to our analysis, *AKT1* displayed the highest weight in the interactive network (Figure 1B).

### Identification of the Shared KEGG Pathways of Tanshinone-Targeted Genes and Osteoporosis

**FIGURE 2:**
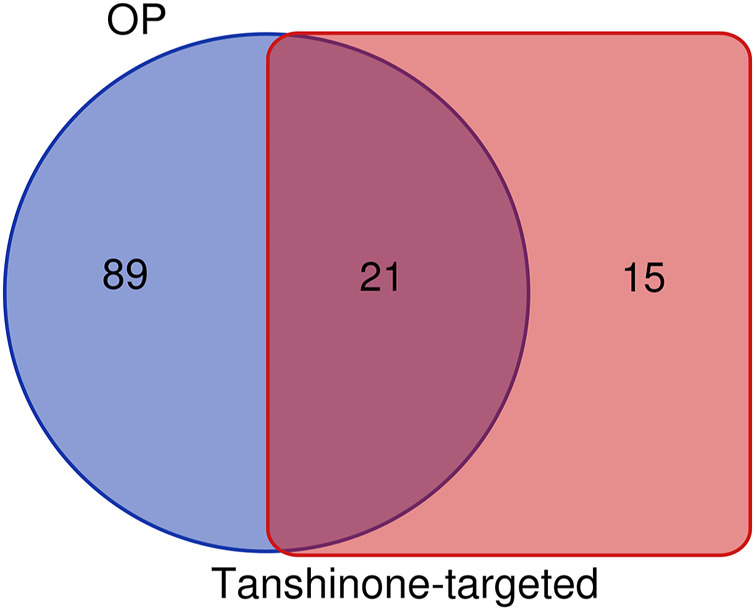
Identification of common KEGG pathways of OP and tomatidine-targeted genes. There are 110 OP related KEGG pathways and 36 tanshinone-targeted genes related KEGG pathways. 21 shared KEGG pathways were identified.

36 KEGG pathways significantly enriched by tanshinone-targeted genes were obtained in DAVID. With miRWalk2.0, 110 human osteoporosis KEGG pathways were retrieved. Finally, 21 common KEGG pathways were identified (Figure 2). The top five KEGG pathways with the smallest *p* values were the small cell lung cancer pathway, Pathway in cancer, Chronic myeloid leukemia pathway, cell cycle pathway, and glioma pathway (Table 2).

**TABLE 2:** Top five KEGG pathway and involved genes.

Term	KEGG pathway	Icariin-Target genes	*p*-Value
hsa05222	Small cell lung cancer pathway	AKT1, CCND1, CDKN1B, XIAP, CDK6, RB1, BIRC3, CDK4, BIRC2, CDK2	3.6E-13
hsa05200	Pathways in cancer	AKT1, CDKN1A, CASP3, CCND1, CDKN2A, CDKN1B, XIAP, CDK6, RB1, BIRC3, CDK4, BIRC2, CDK2	3.6E-11
hsa05220	Chronic myeloid leukemia pathway	AKT1, CDKN1A, CCND1, CDKN2A, CDKN1B, CDK6, RB1, CDK4	4.7E-10
hsa04110	Cell cycle pathway	CDKN1A, CCND1, CDKN2A, CDKN1B, CDK6, RB1, CDK4, CDK2	5.7E-10
hsa05214	Glioma pathway	AKT1, CDKN1A, CCND1, CDKN2A, CDK6, RB1, CDK4	1.4E-8

### Identification of the Hub Genes

**FIGURE 3:**
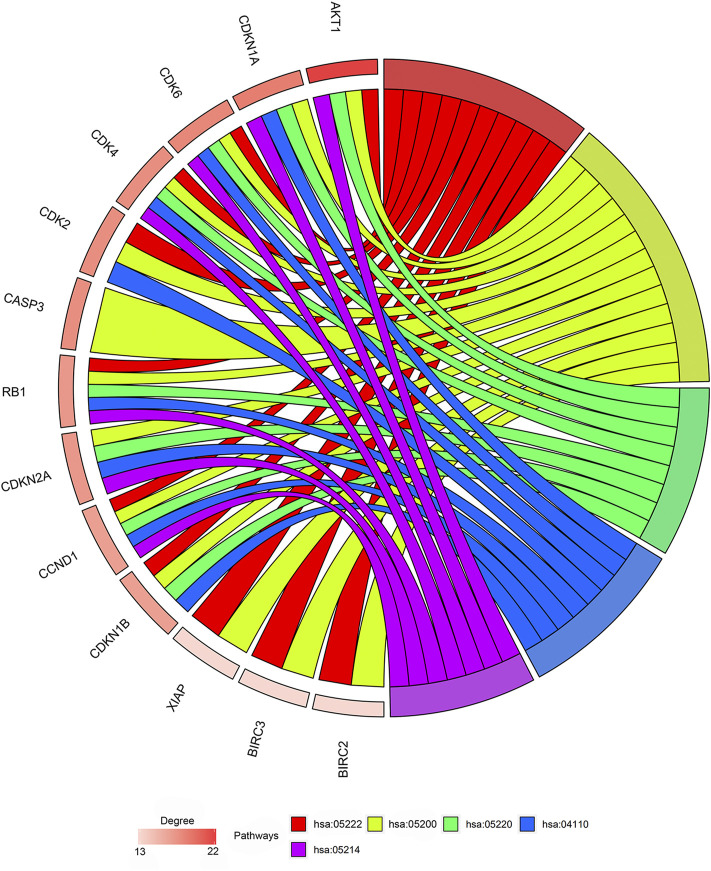
Outcomes of gene enrichment analysis. CCND1, CDK6, RB1, CDK4 were involved in every pathway of the top five. The top three genes with highest degrees are AKT1, CDKN1A, and CDK6. hsa05222: Small cell lung cancer pathway. hsa05200: Pathways in cancer. hsa05220: Chronic myeloid leukemia pathway. hsa04110: Cell cycle pathway. Hsa05214: Glioma pathway.

**FIGURE 4:**
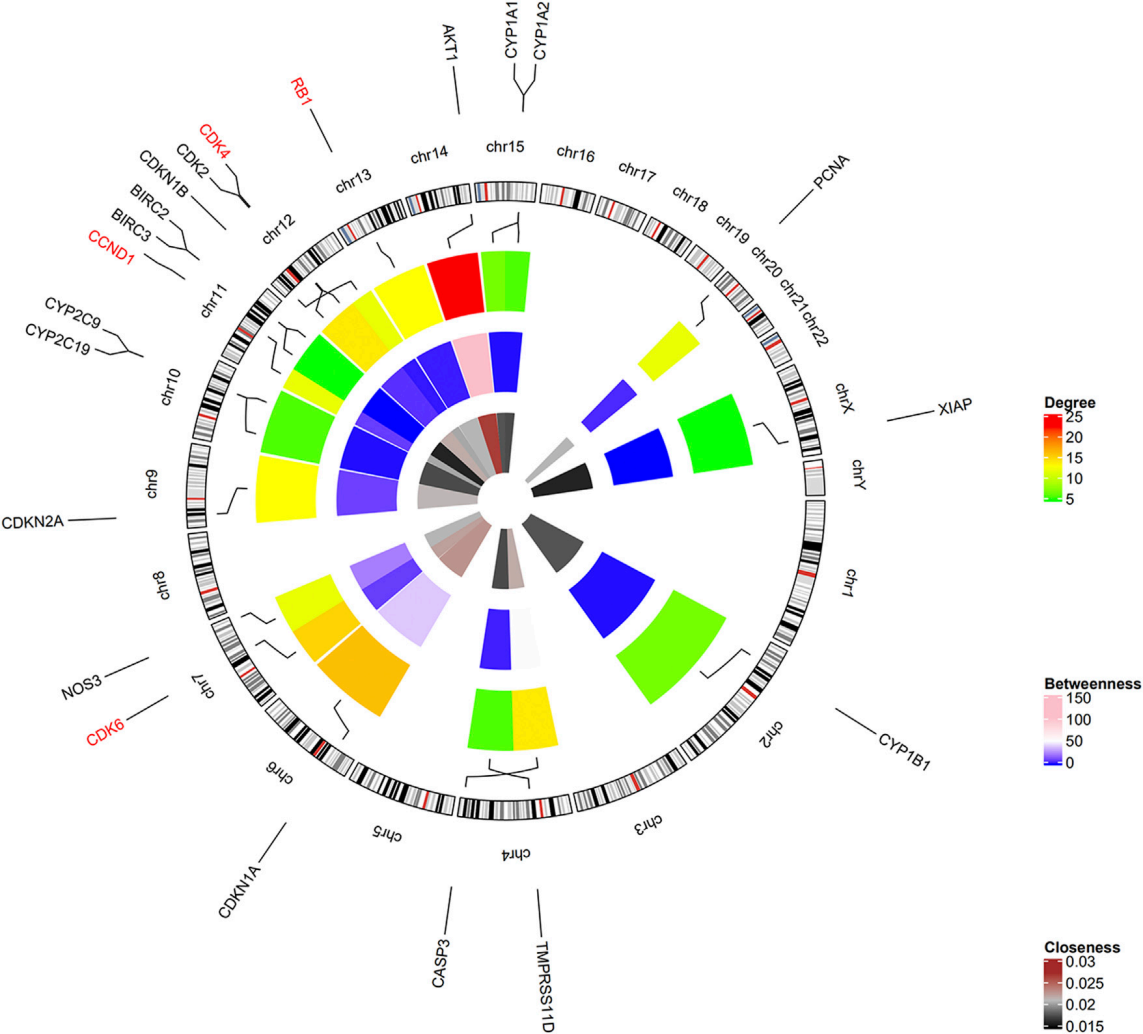
Positions on chromosome and degree centrality information of all the tanshinone-targeted genes. There are three circles of the heatmap. The inner, middle, and outer circles demonstrate closeness, betweenness, and degree information. Hub genes CCND1, CDK6, RB1, CDK4 were located on chr11, chr7, chr13, and chr12, respectively.

Among the 21 tanshinone-targeted genes, *AKT1, CDKN1A, CDK6, CDK4, CDK2, CASP3, RB1, CDKN2A, CCND1, CDKN1B, XIAP, BIRC3* and *BIRC2* were involved in the top five common KEGG pathways (Figure 3). C*CND1, CDK6, RB1, CDK4* were observed to be involved in every common KEGG pathway of the top five. Therefore, they were identified as hub genes. The top three genes with the highest degrees were *AKT1, CDKN1A,* and *CDK6.* The relative positions on the chromosome and degree centrality information of tanshinone-target genes have been shown in Figure 4. *AKT1* displayed the greatest degree, betweenness, and closeness.

### Retrieval of the KEGG Pathways Related to Tanshinone-Targeted Genes

**FIGURE 5:**
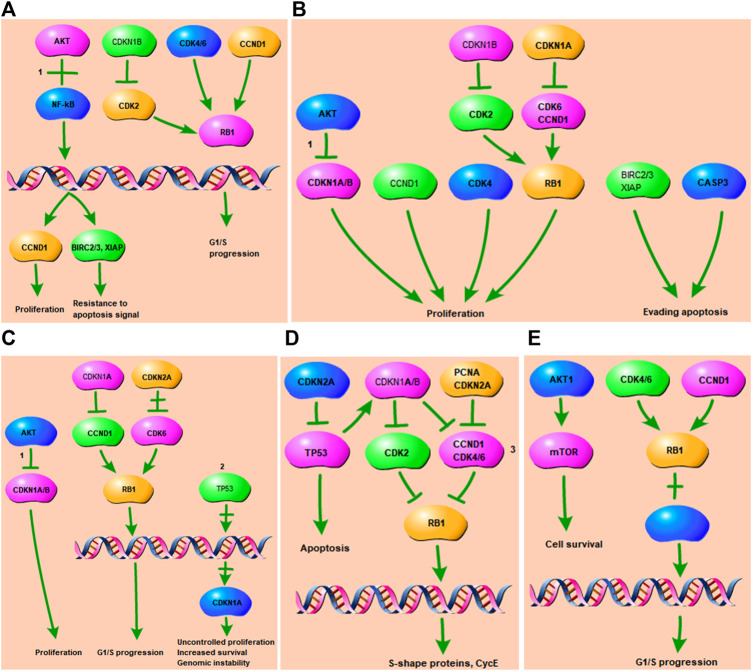
Tanshinone-targeted genes related part of the top two KEGG pathways. 1 PI3K-Akt signaling pathway. 2 p53 signaling pathway. 3 MAPK signaling pathway. **(A)** Tanshinone-targeted genes related part of the Small cell lung cancer pathway. PI3K-Akt signaling pathway is involved in the process, resulting in proliferation, resistance to apoptosis signal, G1/S progression; **(B)** Tanshinone-targeted genes related part of the Pathways in cancer. PI3K-Akt signaling pathway is involved in the process, resulting in proliferation, evading apopsis. **(C)** Tanshinone-targeted genes related part of the Chronic myeloid leukemia pathway. PI3K-Akt and p53 signaling pathway are involved in the process, resulting in proliferation, uncontrolled proliferation, G1/S progression, genomic instability, increased survival. **(D)** Tanshinone-targeted genes related part of the Cell cycle pathway. MAPK signaling pathway is involved in the process, resulting in apoptosis, S-shape proteins, CycE. **(E)** Tanshinone-targeted genes related part of the Glioma pathway, resulting in cell survival and G1/S progression.

The part of the top 5 shared KEGG pathways that can be targeted by tanshinone have been shown in Figure 5. The downstream cellular responses included uncontrolled proliferation, increased survival, resistance to apoptosis signal, genomic instability, G1/S progression, S-shape proteins, CycE. Thus, the results indicated that tanshinone was involved in a variety of cellular activities through affecting PI3K/Akt signaling, p53 and MAPK signaling pathways.

### Tanshinone (Tan) Promoted MSCs Survival *via* Modulating *AKT1* Expression

**FIGURE 6:**
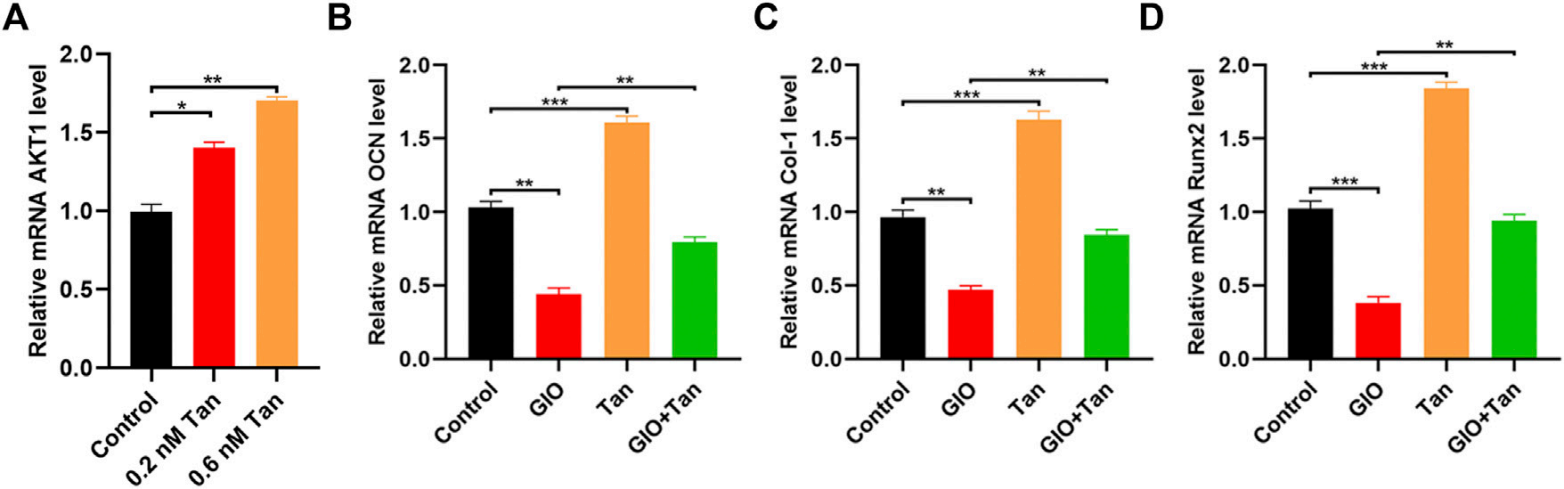
Tanshinone alleviates osteoporosis by targeting AKT1. MSCs were treated with PBS, 0.2 nM Tanshinone, and 0.6 nM Tanshinone **(A)** The expression of AKT1 was assessed by qRT-PCR; **(B–D)** qRT-PCR analysis for the osteogenesis-related mRNAs (OCN, Col-1, Runx2) among the four groups. *p < 0.05, **p < 0.01, ***p < 0.001.

First, the levels of *AKT1* was measured by qRT-PCR, and the result indicated that tanshinone could promote the expression of *AKT1* (Figure 6A). Next, to explore the effect of tanshinone on the osteogenic ability of MSCs, the expression of different osteogenesis-related mRNAs *OCN*, *Col-1*, and *Runx2* were analyzed in the tanshinone-treated cells, and the result indicated a positive impact of the drug on the osteogenesis (Figures 6B–D). Taken together, all these results demonstrated that tanshinone was able to promote osteogenesis *via* activation of *AKT1*.

### *In vivo* Protective Effect of Tanshinone on Osteoporosis

**FIGURE 7:**
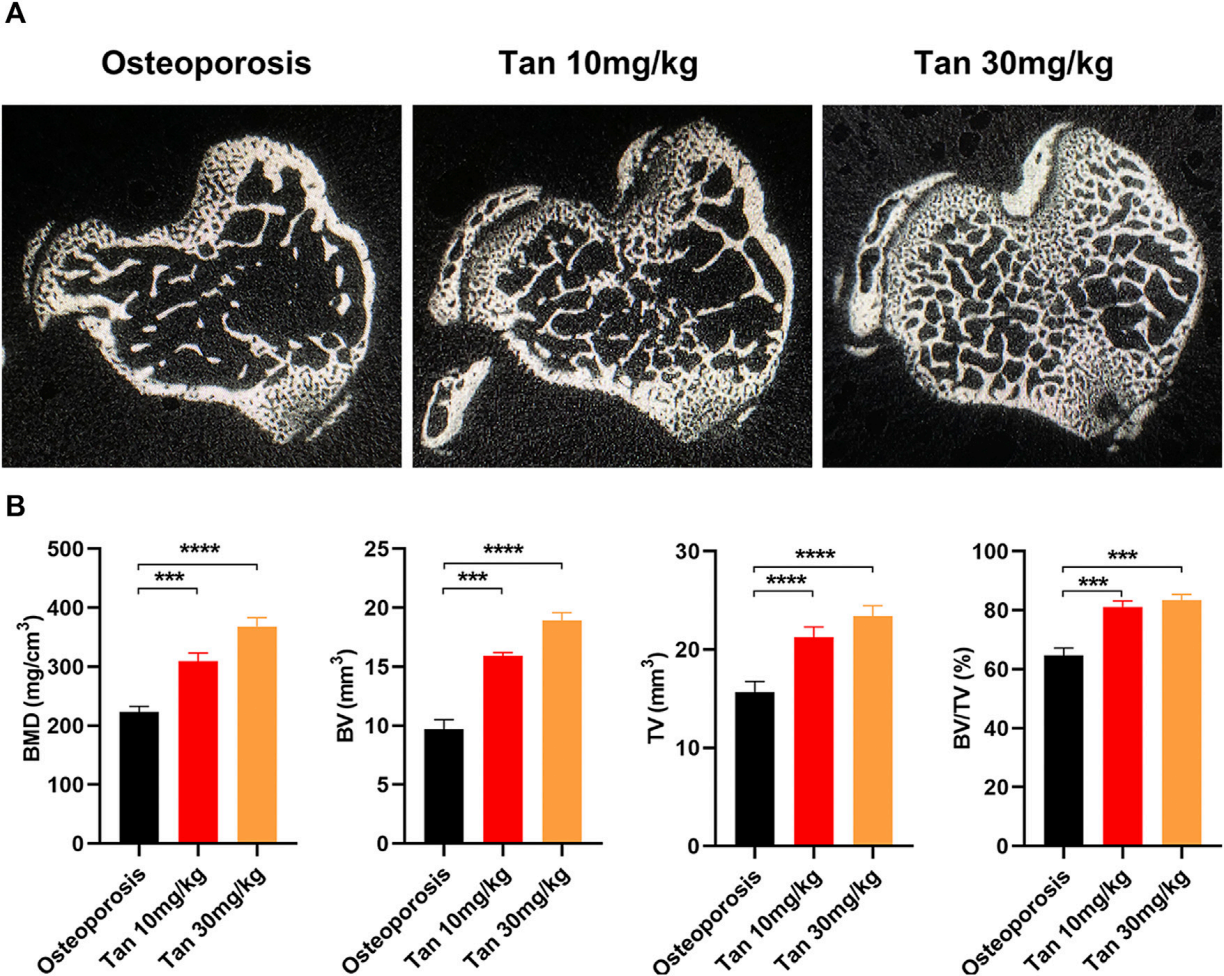
Tanshinone potentially protecting from osteoporosis *in vivo* **(A)** The cross section of bone samples among the three groups; **(B)** BMD, BV, TV, amd BV/TV results were analyzed after receiving the three different treatments for 10 weeks *n* = 10 mice/group. Data are means ± SD of triplicate experiments. *p < 0.05, **p < 0.01, ***p < 0.001.

The effect of tanshinone on osteoporosis was also validated in an *in vivo* mouse model. Consistent with the findings of *in vitro* experiment, BMD, BV/TV, BV, and TV were significantly increased in tanshinone treated group as compared to the osteoporosis group (Figure 7).

## Discussion

Bones play an important role in the protection of internal organs with both rigidity and toughness. The repeated bone reconstruction process primarily maintains integrity and mechanical stress of bone. Bone reconstruction includes resorption of old bone and formation of new bone, which are generally coupled in time and space. Bone reconstruction occurs every moment, throughout the life of a mammal, and the whole body bones are renewed every 10 years (Szulc, 2018).

Bone reconstruction is orchestrated by osteoblast, osteocyte and osteoclast mutually (Xiong et al., 2020b). Osteoblasts produce type I collagen to form a mineralized matrix. The old bone resorption caused by osteoclasts is replaced by new bone formation promoted by osteoblasts. Osteocytes are ultimately differentiated by osteoblasts and embedded in the mineralized matrix (Florencio-Silva et al., 2015). Bone cells can perceive new signals whenever there are changes in the external environment of cells, communicate with osteoblasts and osteoclasts on the bone surface, guide osteoclasts and osteoblasts to the reconstruction site, regulate bone reconstruction, and thus effectively promote repair of microfractures (Buck and Dumanian, 2012). In adolescents, the rate of bone formation has been found to be greater than the resorption. The bones are constantly built, shaped and reconstructed, which can lead to an increase in bone mass. Bone resorption and formation are balanced in adulthood to maintain bone mass. In the elderly, bone resorption rate is greater than formation rate, and osteoporosis begins to occur. In osteoporosis patients, bone mass and strength decreases significantly whereas fracture risk can potentially increase (Feng and McDonald, 2011). Senile osteoporosis presents low bone turnover, but the ratio of bone resorption/bone formation increases, thus leading to progressive bone loss. At the same time, aging and estrogen deficiency can cause the immune system to initiate pro-inflammatory reactions and thereby affect bone reconstruction. Vitamin D deficiency and negative calcium balance in the elderly can also lead to the secondary hyperparathyroidism and affect bone mass (Feng and McDonald, 2011).

The bone reconstruction is in a dynamic equilibrium state under the action of the various systemic or local bone growth regulators. A variety of signal transduction pathways have been reported to be involved in regulating bone reconstruction, such as Hedgehog, MAPK, Notch, RANKL/RANK/OPG, PI3K/Akt, and Wnt/*β*-catenin pathways (Boyce and Xing, 2007; Greenblatt et al., 2010; Regan and Long, 2013; Xi et al., 2015; Yang et al., 2015; Duan and Bonewald, 2016; Mi et al., 2020b). In our study, we found that tanshinone could exhibit a variety of cellular activities through modulating MAPK, PI3K/Akt, and p53 signaling pathways. In the tanshinone-targeted genes network, AKT1 displayed the greatest degree, betweenness, and closeness. Therefore, for the further validation experiments, it was demonstrated that tanshinone could modulate *AKT1* gene expression, promote the MSCs proliferation, and suppress their apoptosis. These findings indicated that tanshinone might improve osteoporosis by targeting AKT1.

There are some limitations associated with this study. Firstly, *in vivo* experiments were not performed in this study. Secondly, the enrichment test of AKT1 in different osteoporosis subtypes was not studied.

## Conclusion

Taken together, our findings indicate that tanshinone can alleviate osteoporosis, which was potentially mediated through modulation of the AKT1 expression. Tanshinone could thus serve as a promising agent for the treatment of osteoporosis.

## Data Availability

The raw data supporting the conclusion of this article will be made available by the authors, without undue reservation.
